# Association of Genetic Variants at *TRPC6* With Chemotherapy-Related Heart Failure

**DOI:** 10.3389/fcvm.2020.00142

**Published:** 2020-08-13

**Authors:** Nadine Norton, Julia E. Crook, Liwei Wang, Janet E. Olson, Jennifer M. Kachergus, Daniel J. Serie, Brian M. Necela, Paul G. Borgman, Pooja P. Advani, Jordan C. Ray, Carolyn Landolfo, Damian N. Di Florio, Anneliese R. Hill, Katelyn A. Bruno, DeLisa Fairweather

**Affiliations:** ^1^Department of Cancer Biology, Mayo Clinic, Jacksonville, FL, United States; ^2^Department of Health Sciences Research, Mayo Clinic, Jacksonville, FL, United States; ^3^Department of Health Sciences Research, Mayo Clinic, Rochester, MN, United States; ^4^Department of Hematology and Oncology, Mayo Clinic, Jacksonville, FL, United States; ^5^Department of Cardiovascular Medicine, Mayo Clinic, Jacksonville, FL, United States; ^6^Center for Clinical and Translational Science, Mayo Clinic, Jacksonville, FL, United States

**Keywords:** anthracycline, doxorubicin, trastuzumab, breast cancer, cardiomyopathy, cardiotoxicity, TWAS, GsMTx-4

## Abstract

**Background:** Our previous GWAS identified genetic variants at six novel loci that were associated with a decline in left ventricular ejection fraction (LVEF), *p* < 1 × 10^−5^ in 1,191 early breast cancer patients from the N9831 clinical trial of chemotherapy plus trastuzumab. In this study we sought replication of these loci.

**Methods:** We tested the top loci from the GWAS for association with chemotherapy-related heart failure (CRHF) using 26 CRHF cases from N9831 and 984 patients from the Mayo Clinic Biobank which included CRHF cases (*N* = 12) and control groups of patients treated with anthracycline +/– trastuzumab without HF (*N* = 282) and patients with HF that were never treated with anthracycline or trastuzumab (*N* = 690). We further examined associated loci in the context of gene expression and rare coding variants using a TWAS approach in heart left ventricle and Sanger sequencing, respectively. Doxorubicin-induced apoptosis and cardiomyopathy was modeled in human iPSC-derived cardiomyocytes and endothelial cells and a mouse model, respectively, that were pre-treated with GsMTx-4, an inhibitor of TRPC6.

**Results:** *TRPC6 5*′ flanking variant rs57242572-T was significantly more frequent in cases compared to controls, *p* = 0.031, and rs61918162-T showed a trend for association, *p* = 0.065. The rs61918162 T-allele was associated with higher *TRPC6* expression in the heart left ventricle. We identified a single *TRPC6* rare missense variant (rs767086724, N338S, prevalence 0.0025% in GnomAD) in one of 38 patients (2.6%) with CRHF. Pre-treatment of cardiomyocytes and endothelial cells with GsMTx4 significantly reduced doxorubicin-induced apoptosis. Similarly, mice treated with GsMTx4 had significantly improved doxorubicin-induced cardiac dysfunction.

**Conclusions:** Genetic variants that are associated with increased *TRPC6* expression in the heart and rare *TRPC6* missense variants may be clinically useful as risk factors for CRHF. GsMTx-4 may be a cardioprotective agent in patients with *TRPC6* risk variants. Replication of the genetic associations in larger well-characterized samples and functional studies are required.

## Introduction

Cardiomyopathy is a major comorbidity for cancer patients treated with anthracyclines and/or trastuzumab (1–7). Doxorubicin-related heart failure (CRHF) is dose dependent at doses >250 mg/m^2^ (8); However, even at “safe” doses of <250 mg/m^2^ around 2% of patients develop heart failure (HF), while 26% of patients develop mild left ventricular (LV) dysfunction within 6 months (9) and these cardiac events occur more often in patients who receive anthracycline plus trastuzumab (1, 5). Additionally, mediastinal radiation therapy and the presence of co-morbidities such as hypertension or diabetes may further increase the risk of chemotherapy-related cardiomyopathy and are managed primarily by limiting the cumulative dose of doxorubicin, prompt referral to a cardiologist, and by treatment with standard HF medications. There are no definitive recommendations regarding continuation or discontinuation of chemotherapy in individuals with evidence of cardiac dysfunction and careful consideration of the risks and benefits of therapy is needed (10).

Current risk models are unable to accurately predict which patients will develop HF following chemotherapy and prospective studies that use standard HF medications to reduce the incidence of chemotherapy-related congestive heart failure (CHF) have had limited efficacy (11). Hence, there is a critical need for comprehensive risk prediction models that are able to identify the population most “at risk” in a timely manner, ideally incorporating both clinical and pharmacogenomic risk factors.

To identify novel genes that could promote cardiomyopathy following cancer therapy, we previously completed a genome-wide association study (GWAS) of maximum decline in left ventricular ejection fraction (LVEF) in 1,191 patients treated with doxorubicin +/–trastuzumab in the N9831 clinical trial in early breast cancer patients (12). We used linear regression to identify six novel risk loci associated with the maximum decline in LVEF following cancer therapy: *LDB2* (*p* = 8.9e^−8^), *BRINP1* (*p* = 5.9e^−7^), Chr6 intergenic (*p* = 1.4e^−6^), *RAB22A* (*p* = 5.6e^−6^), *LINC01060* (*p* = 7.7e^−6^), and *TRPC6* (*p* = 7.7e^−6^). Here, we further evaluated these loci to look for replication using CHF as a binary phenotype. We tested for replication using two different control groups with the goal of replication of variants associated specifically with chemotherapy. We looked for convergent evidence of association using transcriptome-wide association and exon sequencing, identifying eQTLs and missense variants in *TRPC6* as putative risk variants for CRHF and used human iPSC-derived cardiomyocytes and endothelial cells to test the inhibition of TRPC6 as a potential therapy for doxorubicin-induced cardiotoxicity.

## Materials and Methods

### Patient Populations (Figure 1)

#### N9831 Clinical Trial

Patients gave written, informed consent for participation in the N9831 clinical trial. Both the N9831 trial as well as the current study were approved by the institutional review board at the Mayo Clinic. N9831 was a pivotal clinical trial that led to trastuzumab being used as the standard of care for early HER2+ breast cancer. The study compared adjuvant chemotherapy (doxorubicin and cyclophosphamide followed by paclitaxel) only (Arm A) vs. adjuvant chemotherapy followed by trastuzumab either sequentially (Arm B) or concurrently (Arm C) in patients with operable HER2+ breast cancer (13, 14). Patients received serial echocardiograms (ECHO) or multigated acquisition scans (MUGA) at baseline, 3, 6, and 9 months after registration, and after completion of chemotherapy and some women consented for a follow up evaluation at 6 years. In the trial, cardiac events were defined as symptomatic CHF, definite cardiac death as a result of myocardial infarction, CHF or arrhythmia, or probable cardiac death without documented etiology. The trial found that cardiac events were significantly higher in patients receiving trastuzumab (3%) compared to patients receiving chemotherapy alone (0.6%) at a median follow up of 9.2 years (1).

**Figure 1:**
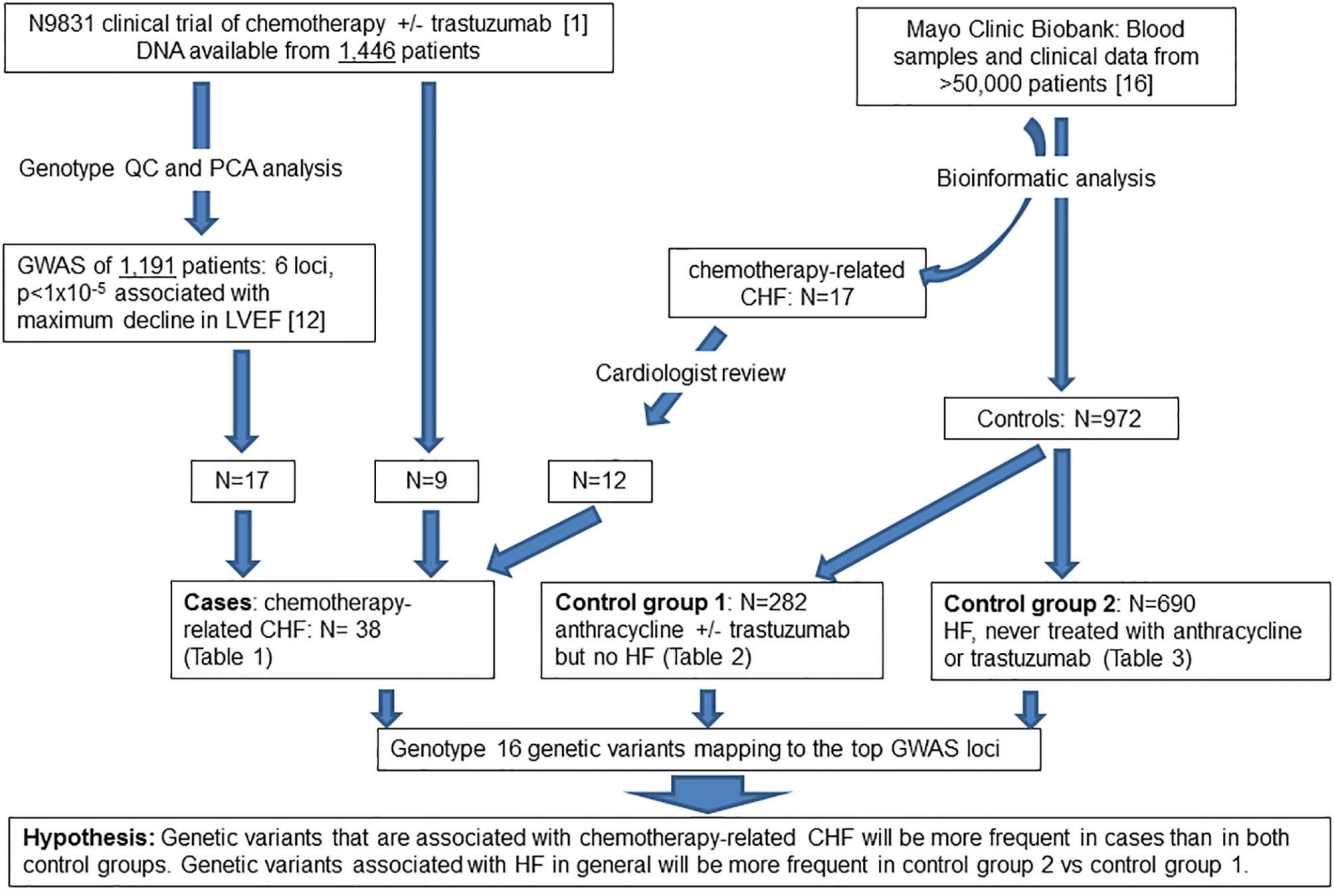
Flow diagram of patient populations and experimental plan. QC, quality control; PCA, principal component analysis; GWAS, genome-wide association study; LVEF, left ventricular ejection fraction; CHF, congestive heart failure, HF, heart failure.

We previously obtained DNA specimens from 1,446 consenting patients in the N9831 trial. Our published GWAS analysis was limited to 1,191 patients (Arm A, *N* = 391 and Arms B and C combined, *N* = 800) after exclusion of 188 patients reported as non-White/Hispanic. Principal components analysis identified a further 27 outliers and 40 patients were missing either baseline or post-treatment LVEF. Thus, in 1,191 patients determined as White non-Hispanic we used linear regression to test for association of the maximum decline in LVEF with each SNP (12). In the current study, prediction of gene expression in N9831 was limited to the same set of patients because the performance of PrediXcan varies substantially among populations, with predictions of gene expression in patients from African Ancestry having the lowest accuracy (15). Subsequent DNA sequencing and genotyping analyses were performed on DNA samples from 26 patients in the N9831 trial that were confirmed to have chemotherapy-related CHF (all female, age at treatment 32–79 years, mean age 55.0 +/−10.3 years). Only 17 of the 26 patients were included in the primary GWAS analysis based on PCA analysis of race and ethnicity. Of the 9 patients that were outliers in PCA analysis, four were self-reported as Black/African American, one Hispanic, one Asian, and four were self-reported as White (Table 1).

**Table 1:** Cardiologist reviewed chemotherapy-related congestive heart failure group demographics, age at chemotherapy and chemotherapy regimen.

	**Male (%)**	**Female (%)**
**RACE**		
White	4 (100)	29 (85.3)
Black/African American	0 (0)	4 (11.8)
Asian	0 (0)	1 (2.9)
Mixed	0 (0)	0 (0)
**ETHNICITY**		
Non-hispanic/latino	4 (100)	33 (97.1)
Hispanic/latino	0 (0)	1 (2.9)
Unknown	0 (0)	0 (0)
**AGE AT CHEMOTHERAPY (YEARS)**		
Range	61–81	32–82
Mean	73.5	58.0
Median	76.0	57.0
Standard deviation	8.7	12.0
**CHEMOTHERAPY REGIMEN**		
RCHOP/CHOP	3 (75)	2 (5.9)
Anthracycline	1 (25)	0 (0)
Anthracycline, cyclophosphamide	0 (0)	2 (5.9)
Anthracycline, taxane, cyclophosphamide	0 (0)	12 (35.3)
Anthracycline, taxane, cyclophosphamide, trastuzumab	0 (0)	17 (50)
Taxane, trastuzumab, pertuzumab	0 (0)	1 (2.9)

*Cases included 38 patients of which 12 were identified in the Mayo Clinic Biobank. Twenty-six were patients in the N9831 clinical trial of early breast cancer. 17/26 patients from the N9831 clinical trial were included in the primary GWAS analysis of decline in LVEF: 7/17 patients were in Arm A (doxorubicin, cyclophosphamide, paclitaxel) and 10 patients were in Arms BC (doxorubicin, cyclophosphamide, paclitaxel, trastuzumab. Nine patients from the N9831 trial were not included in the initial GWAS: 2/9 were in Arm A and 7/9 were in Arms BC. Abbreviations: RCHOP, rituximab, cyclophosphamide, doxorubicin, vincristine sulfate and prednisone*.

#### Mayo Clinic Biobank

The Mayo Clinic Biobank is a collection of blood samples and health related data on over 50,000 Mayo Clinic patients designed to be utilized for numerous research projects that has been described elsewhere (16). Subjects provided written, informed consent that allowed access to all Mayo Clinic health record data. Both the Mayo Clinic Biobank as well as the current study were approved by the institutional review board at the Mayo Clinic. Heart failure and chemotherapy were two important data elements for the identification of study groups. To obtain HF phenotype, we implemented the eMERGE algorithm on all Mayo Clinic Biobank participants to identify patients under a hierarchical outcome of either definite, probable or possible HF, based on the degree of confidence (17). Chemotherapy was then identified from chemotherapy order data as well as unstructured clinical notes, of which the drug names (any type of anthracycline and trastuzumab) were extracted from the “current medication” section using a natural language processing tool, MedXN (18). Using the temporal information associated with HF and chemotherapy from electronic health records (EHRs) of Mayo Clinic Biobank participants, we identified 984 patients enrolled between 4/14/2009 and 11/18/2015 into three study groups.

The first group consisted of 282 patients (male *N* = 45, female *N* = 237) who completed anthracycline and/or trastuzumab and showed no signs of HF (definite, probable or possible) as determined by the eMERGE algorithm (17). Age at treatment ranged from 14 to 90 years (mean 58.7 years, SD 13.7 years, median 59 years). Mean follow-up time was 6.11 years (SD 4.20 years) and median follow-up time was 4.79 years. We further screened this control group by ICD codes for cardiomyopathies. Three patients had a diagnosis of a primary cardiomyopathy, one patient had ischemic cardiomyopathy and one patient had an unspecified adverse effect prior to anthracycline. Race and ethnicity were self-reported as 95% White, 1% African American, 1% Asian, 3% mixed race, and 0% Hispanic/Latino. Demographics, site of cancer, and age at treatment for males and females are shown in Table 2.

**Table 2:** Biobank control group 1 demographics, age at treatment and sites of cancer.

	**Male (%)**	**Female (%)**
**RACE**		
White	41 (91.1)	227 (96)
Black/African American	0 (0)	2 (0.8)
Asian	1 (2.2)	2 (0.8)
Mixed	3 (6.7)	6 (2.6)
**ETHNICITY**		
Non-hispanic/latino	44 (97.8)	234 (98.3)
Hispanic/latino	0 (0)	0 (0)
Unknown	1 (2.2)	4 (0.17)
**AGE AT CHEMOTHERAPY (YEARS)**		
Range	26–90	14–90
Mean	65.7	57.4
Standard deviation	13.0	13.5
Median	66.0	57.0
SITE OF CANCER^*^		
Breast	3 (5.1)	168(67.5)
Blood	24 (40.7)	20 (8.0)
Sarcoma	8 (13.6)	14 (5.6)
Gastrointestinal	6 (10.2)	1 (0.4)
Ovarian	0 (0)	18 (7.2)
Lung	3 (5.1)	5 (2.0)
Bladder	2 (3.4)	2 (0.8)
Skin	1 (1.7)	3 (1.2)
Prostate	3 (5.1)	0 (0)
Unknown	2 (3.4)	9 (3.6)
Neuroendocrine	1 (1.7)	2 (0.8)
Head/neck	1 (1.7)	1 (0.4)
Testicular	2 (3.4)	2 (0.8)
Liver	3 (5.1)	3 (1.2)
Metastasis to lymph node axilla and upper limb	0 (0)	1 (0.4)

*Control group one included 282 patients who completed either anthracycline and/or trastuzumab therapy without a diagnosis of heart failure (either: definite, probable or possible by the eMERGE algorithm)*.

* *16 patients (9 male and 7 female had cancer diagnoses at multiple sites)*.

The second group consisted of 690 patients with definite or probable HF (possible heart failure was excluded) who had never received anthracycline or trastuzumab (male *N* = 384, female *N* = 306, age at HF 22–101 years, mean age 75.1 +/– 10.8 years) determined by the same algorithm. Race and ethnicity were self-reported as 95% White, 0.6% African American, 0.1% Asian, 1.2% mixed race, 0.3% Hispanic/Latino, and 3.2% unknown/not reported. Demographics, age at HF, and site of any cancers prior to HF diagnosis for males and females are shown in Table 3. Fifty-seven percentage of patients in this group had a diagnosis of cancer prior to the diagnosis of HF, the most common site being skin (*N* = 220).

**Table 3:** Biobank control group 2 demographics, age at heart failure and sites of prior cancer.

	**Male (%)**	**Female (%)**
**RACE**		
White	365 (95.1)	290 (94.8)
Black/African American	3 (0.8)	1 (0.3)
Asian	1 (0.3)	0 (0.0)
Mixed	3 (0.8)	5 (1.6)
Unknown	12 (3.1)	10 (3.3)
**ETHNICITY**		
Non-hispanic/latino	372 (96.9)	294 (96.1)
Hispanic/latino	0 (0)	2 (0.7)
Unknown	12 (3.1)	10 (3.2)
**AGE AT HEART FAILURE (YEARS)**		
Range	22–97	31–101
Mean	74.2	76.2
Standard deviation	10.7	11.0
median	76	78
**SITE OF CANCER**		
Skin	129 (42.7)	91 (42.3)
Prostate	73 (24.2)	0 (0)
Breast	0 (0)	45 (20.9)
Blood	19 (6.3)	16 (7.4)
Gastrointestinal	23 (7.6)	9 (4.2)
Kidney	13 (4.3)	4 (1.9)
Lung	14 (4.6)	3 (1.4)
Bladder	11 (3.6)	5 (2.3)
Ovarian	0 (0)	11 (5.2)
Neuroendocrine	10 (3.3)	2 (0.9)
Sarcoma	3 (1.0)	5 (2.3)
Endometrial	0 (0)	7 (3.3)
Cervical	0 (0)	6 (2.8)
Pancreas	3 (1.0)	2 (0.9)
Brain	1 (0.3)	2 (0.9)
Head/neck	2 (0.7)	4 (1.9)
Uterus	0 (0)	2 (0.9)
Heart	0 (0)	1 (0.5)
Lynch syndrome	1 (0.3)	0 (0)

*Control group 2 included 690 patients that had never received anthracycline or trastuzumab and were determined by the eMERGE algorithm to have definite or probable heart failure. 392 (57%) of patients in this group had a cancer diagnosis prior to the diagnosis of heart failure, and 100/392 patients had more than one cancer diagnosis*.

We identified an additional 17 chemotherapy cases with HF from the Mayo Clinic Biobank with an outcome of definite or probable HF determined by the eMERGE algorithm that were then assessed by an independent cardiologist (Landolfo) through examination of medical records. 5/17 patients with HF that were determined to have valve or ischemic disease were eliminated from genetic analyses. 12/17 cases were determined to be the result of anthracycline and/or trastuzumab therapy (male *N* = 4, female *N* = 8, age at anthracycline treatment ranged from 49 to 82 years, mean 70.7 years, SD 10.0 years, median 73.5 years) (Table 1). Stored DNA from the Mayo Clinic Biobank was genotyped on all 984 patients (males *N* = 433, females *N* = 551).

### Laboratory Methods

#### Sanger Sequencing

PCR primers were designed for 13 exons and flanking intronic sequence of *TRPC6*. Exons of 38 patients with chemotherapy-related CHF (26 from N9831 and 12 from the Mayo Clinic Biobank) were screened for *TRPC6* variants by bi-directional sequencing using Big-Dye (v3.1) terminator chemistry and an ABI3730 sequencer according to the manufacturer's instructions (Applied Biosystems). Missense variants identified by Sanger Sequencing were also confirmed by genotyping on the Sequenom platform described below.

#### SNP Genotyping

*SNP genotyping:* 16 SNPs were genotyped using the Sequenom platform. These SNPs included the most significantly associated SNPs reported in our initial GWAS at *p* < 1 × 10^−5^: *TRPC6* rs77679196; *BRINP1* rs62568637; *LDB2* rs55756123; *RAB22A* rs707557; intergenic 6p22.3 rs4305714. For *TRPC6*, we also genotyped an additional 11 SNPs, five of which mapped to the 5′ flanking region (rs11224819, rs5724572, rs1938858, rs11224953, rs11224983) and were significantly associated at *p* < 1 × 10^−5^ in the imputation analysis from the primary GWAS, four of which were eQTLs in GTEX tissue from heart left ventricle or artery tibial tissue *p* < 1 × 10^−5^ (rs61918162, rs2513192, rs12280648, rs4509717), two missense variants (rs36111323, A404V, a common variant associated in our initial GWAS using a logistic regression model), and a rare variant (rs767086724, N338S) identified by Sanger sequencing of 38 patients with chemotherapy-related CHF.

#### Genotyping Quality Control

Nine hundred and eighty-four DNA samples from the Mayo Clinic Biobank were plated into 11 96-well-plates. Each 96-well-plate included two duplicate samples from the next plate and two blank wells used for negative controls. Genotyping was 100% concordant for 22 duplicate DNA samples across 16 SNPs (352 genotypes).

#### Cell-Based Assays

Human iPSC-derived cardiomyocytes (iCell^®^ cardiomyocytes^2^) and endothelial cells (iCell^®^ endothelial cells) were purchased from Cellular Dynamics Inc. (Madison, WI) and grown as per the manufacturer's instructions and our previous publication (19). Cardiomyocytes were exposed to doxorubicin (Selleckchem, Houston, TX) at final concentrations of 0.25, 0.5, 1, 2.5, 5, and 10 μM for 24 h prior to examining apoptosis. Endothelial cells were exposed to doxorubicin at a final concentration of 1 μM. Cells were treated with GsMTx-4, purchased from CSBio (Menlo Park, CA) at a final concentration of 5 μM, 4 h prior to doxorubicin exposure. Apoptosis was measured with the Caspase-Glo 3/7 assay (Promega Corporation, Madison WI) as per the manufacturer's instructions. Each drug combination was performed in replicates of four or eight.

#### Animal Experiments

Animal protocols were performed according to NIH guidelines with approval from the Institutional Animal Care and Use Committee, and the Biosafety Committee at Mayo Clinic. Mice were maintained under pathogen-free conditions in the animal facility at the Mayo Clinic, fed standard chow, and housed in animal rooms where the temperature was monitored. Male 8 week old adult wild type BL6.129 mice (*n* = 7–8/group) were obtained from the Jackson Laboratory (Bar Harbor, ME) (catalog #101045) and exposed to 6 doses of 4 mg/kg doxorubicin (Selleckchem, Houston, TX) or saline/DMSO control intraperitoneally (ip) on days 1, 3, 5, 8, 10, 12 (cumulative dose 24 mg/kg), and hearts were evaluated for fibrosis/remodeling using histology, cardiac function using echocardiography to determine left ventricular ejection fraction (LVEF) and echocardiography-derived global longitudinal strain (GLS) on day 21 according to (20–22). The Trpc6 inhibitor GsMTx-4 (10 mg/kg) (CSBio, Menlo Park, CA) was initially diluted in a small volume of sterile de-ionized reverse osmosis water and then in a larger volume of saline vs. saline control was administered on day 0, 7, 14 ip. Echocardiography was performed on living animals under isoflurane inhalation. Cardiac function of the left ventricle was assessed using echocardiography and echocardiography-derived strain based speckle tracking in long-axis view as described in (20, 22). The extent of fibrosis was determined histologically from 5 μ sections of the heart that were stained with Masson's trichrome as the percentage of the heart section with fibrosis normalized to the overall size of the heart section according to (21).

### Statistical Analysis

#### Gene Expression in N9831 Patients and Gene-Based Association Testing

Linear regression was used to assess association of gene variants with change in LVEF (lowest recorded LVEF—baseline LVEF) as the response variable; these models were adjusted for age, baseline LVEF, anti-hypertensive medications, and the first two principal components of the genotypes in the 800 patients in the N9831 trial who received combination chemotherapy and trastuzumab as per our initial GWAS (12). We used PrediXcan (23) to perform association testing of the change in LVEF with the estimated heritable gene expression levels in left ventricular tissue of patients in N9831, using genotype and expression data from 159 individuals in the GTEX database as a training set.

#### Association Testing of Variants With Chemotherapy-Related Heart Failure

The Mayo Clinic Biobank patients were divided into two control groups. The first group consisted of 282 patients who completed chemotherapy and showed no signs of heart failure. The second control group consisted of 690 patients with heart failure of any cause with no prior exposure to anthracycline or trastuzumab. Cases consisted of patients with CRHF, (Mayo Clinic Biobank *N* = 12, and N9831 *N* = 26). We assumed that *TRPC6* variants that are uniquely associated with CRHF would be more (or less) frequent in the cases than in either of the control groups. Our null hypothesis is that *TRPC6* variants have frequency in the CRHF patients that is the same as the frequency in at least one of the control groups. We used logistic regression analyses with the binary outcome being the presence of a *TRPC6* variant, and included 2 parameters corresponding to the odds of having the variant in the case group compared to each of the two control groups. We optimized the likelihood under both scenarios to obtain the likelihood ratio test (LRT) statistic and estimated the distribution of the test statistic via bootstrap simulation. A two-sided test was conducted via combining the results of two one-sided bootstrap tests via the min-P method: the two-sided *p*-value is calculated as twice the smaller of the one-sided *p*-values.

#### Association Testing of Heart Failure

To further explore whether variants were risk factors for HF in general, we performed association testing with the Mayo Clinic Biobank patients with heart failure who had never been exposed to chemotherapy (*N* = 690, defined as cases) and patients with prior exposure to chemotherapy who did not have HF (*N* = 282, defined as controls). We tested for association of each variant using logistic regression, adjusting for sex, and use of anti-hypertensive medications. These analyses were performed in plink 1.07 under an additive model.

#### Cell Based Assays

Comparisons of doxorubicin-induced apoptosis in cardiomyocytes and endothelial cells exposed to doxorubicin with or without GsMTx-4 pretreatment were performed in GraphPad Prism using Student's *T*-test.

#### Animal Experiments

A non-parametric test for differences in means (ANOVA or Kruskal-Wallace) were conducted and Bonferroni adjustment (*p* < 0.017 regarded as significant; alpha for 3 tests = 0.05/3 = 0.017) for multiple comparisons between individual groups (Sidak or Mann-Whitney) with N/group = 7–8 mice.

## Results

### Imputed *TRPC6* Gene Expression Levels in the Left Ventricle of the Heart Are Associated With a Decline in LVEF in Breast Cancer Patients Who Were Treated With Both Chemotherapy and Trastuzumab

**Figure 2:**
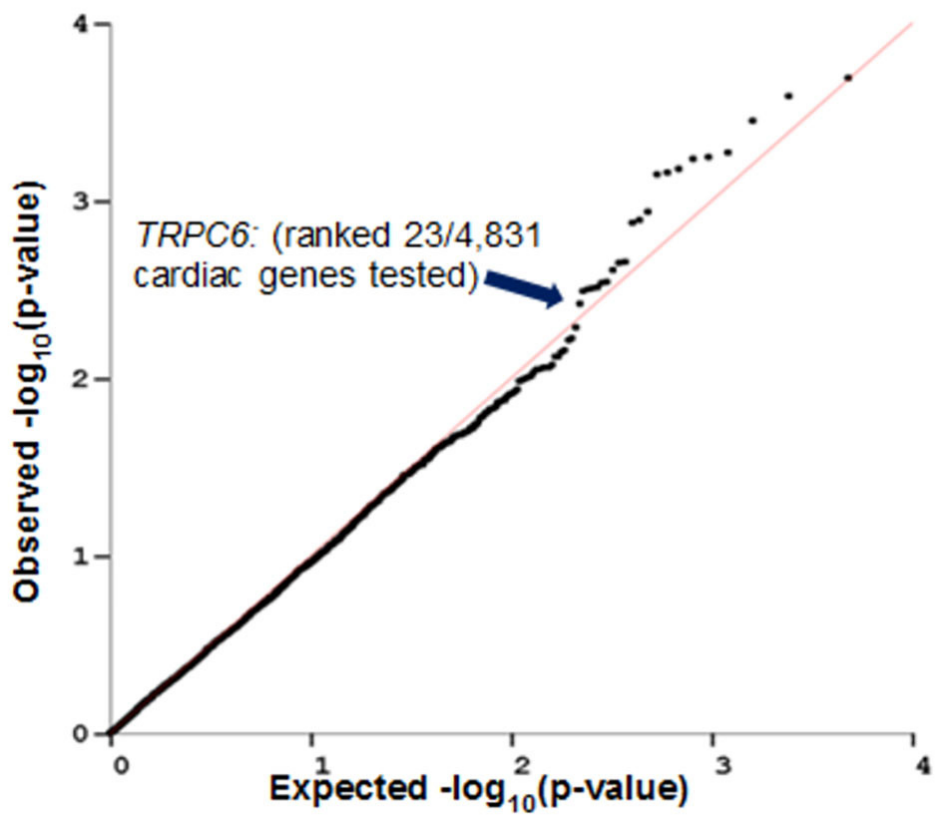
Imputed *TRPC6* gene expression levels in heart left ventricle are associated with decline in LVEF in breast cancer patients who were treated with anthracycline and trastuzumab. PrediXcan was used to impute gene expression in left ventricular cardiac tissue of patients from N9831 and test for association analysis of gene expression with maximum decline in LVEF. Gene expression was imputed for 4,853 genes. *TRPC6* (one of the top hits in the primary GWAS analysis) ranked 23/4,853 genes.

PrediXcan (23) was used to impute the heritable portion of gene expression in left ventricular cardiac tissue of patients from N9831 and test for association of imputed gene expression with maximum decline in LVEF. A QQ plot of the observed vs. expected *p*-values of cardiac gene expression and decline in LVEF in N9831 patients is shown in Figure 2. Gene expression was imputed for 4,853 genes. None of the 4,853 genes reached the criteria for genome-wide significance. Of the top six loci in the primary GWAS (12), imputed expression of *TRPC6* was associated with a decline in LVEF, *p* = 0.005, ranking 23/4,853 genes. None of the other top GWAS loci were able to be imputed in left ventricular cardiac tissue of patients. The top 50 genes from this analysis are shown in Supplementary Table 1.

### *TRPC6* 5′ Flanking Variant rs57242572 and *LDB2* rs55756123 Are Associated With Chemotherapy-Related Heart Failure

Following our finding that the heritable portion of *TRPC6* gene expression in tissue from the LV is associated with a decline in LVEF in patients treated with chemotherapy and trastuzumab from the N9831 clinical trial, we sought to validate this finding in additional samples and to determine if the association was a direct result of chemotherapy or whether genetic variants at the *TRPC6* locus simply increased a patient's risk of HF in general. For comparison, we used two different control groups from patients in the Mayo Clinic Biobank and compared allele frequencies with those of our sample of patients with CRHF. Genotype counts for each SNP and each control and case group are shown in Supplementary Table 2.

Control group one consisted of patients who completed chemotherapy and did not have a diagnosis of HF at any time. The second control group consisted of patients with HF of any cause who had no prior exposure to anthracycline or trastuzumab. Cases consisted of 38 patients with CRHF of which 12 patients were from the Mayo Clinic Biobank and 26 patients were from the N9831 clinical trial (17/26 patients were included in the primary GWAS analysis).

We assumed that frequencies of *TRPC6* variants that were uniquely associated with CRHF would differ between cases compared with both of the control groups. We found evidence of association for rs57242572, *p* = 0.031 and a trend for association with rs61918162, *p* = 0.065 (Table 4). rs61918162 was specifically selected for genotyping because in the GTEx database the T allele was strongly associated with higher gene expression of *TRPC6* in tissue from the left ventricle of the heart, *p* = 8 × 10^−7^. The rs61918162-T allele was detected more frequently in patients with CRHF, suggesting that higher gene expression levels of *TRPC6* are associated with HF from anthracycline or anthracycline plus trastuzumab. rs767086724, a rare missense (N338S) variant was also associated with CRHF, *p* = 0.014, and is discussed in the next section.

**Table 4:** Association analysis of chemotherapy-related congestive heart failure.

**SNP**	**Locus**	**Minor allele**	**Chemo No HF (*N* = 282)**	**No Chemo + HF (*N* = 690)**	**Chemo + HF (*N* = 38)**	***p*-value**
rs36111323	*TRPC6*	A	0.082	0.144	0.066	0.648
rs767086724	*TRPC6*	C	0.000	0.000	0.013	**0.014**
rs11224819	*TRPC6*	C	0.121	0.082	0.079	0.325
rs4509717	*TRPC6*	G	0.478	0.415	0.368	0.090
rs12280648	*TRPC6*	G	0.247	0.259	0.237	0.797
rs77679196	*TRPC6*	A	0.007	0.004	0.026	0.126
rs57242572	*TRPC6*	T	0.013	0.015	0.053	**0.031**
rs2513192	*TRPC6*	A	0.259	0.283	0.276	0.725
rs1938858	*TRPC6*	T	0.188	0.192	0.224	0.405
rs61918162	*TRPC6*	C	0.394	0.354	0.276	**0.065**
rs11224953	*TRPC6*	G	0.051	0.037	0.053	0.866
rs11224983	*TRPC6*	A	0.047	0.038	0.066	0.451
rs55756123	*LDB2*	T	0.007	0.006	0.040	**0.030**
rs4305714	intergenic 6p22.3	T	0.216	0.222	0.263	0.302
rs62568637	*BRINP1*	A	0.013	0.009	0.026	0.328
rs707557	*RABB22A*	T	0.399	0.431	0.355	0.473

*Minor allele frequencies are shown in each of three groups: Chemo No HF = treated with anthracycline or anthracycline plus trastuzumab and no diagnosis of heart failure (definite, probable or possible); No Chemo + HF = never received anthracycline or trastuzumab and diagnosis of heart failure; Chemo + HF = cardiologist reviewed diagnosis of chemotherapy-related congestive heart failure following anthracycline or anthracycline plus trastuzumab. SNP, single nucleotide polymorphism*.

Our SNP panel also included the sentinel SNPs from five loci reported in our initial GWAS analysis of decline in LVEF in response to chemotherapy plus trastuzumab (*TRPC6, BRINP1, LDB2, RAB22A*, intergenic region 6p22.3). rs55756123, located in the 3′ flanking region of *LDB2*, was also significantly associated with CRHF relative to the two control groups, *p* = 0.030.

### *TRPC6* Rare Missense Variants in Patients With Chemotherapy-Related Heart Failure

Given the indirect nature of GWAS, the known association of rare missense variants in *TRPC6* with the genetic form of chronic kidney disease, focal segmental glomerular sclerosis (FSGS) (24, 25), and the fact that FSGS can result from exposure to anthracyclines (26), we sequenced the coding regions of *TRPC6* in 38 patients with CRHF. None of the confirmed causative FSGS mutations were identified in patients with CRHF.

We observed three missense variants in the 38 CRHF cases: rs3802829 (P15S), rs36111323 (A404V), and rs767086724 (N338S). P15S and A404V are common with minor allele frequencies of 0.10 and 0.12 in European populations in GnomAD while N338S is very rare.

**Figure 3:**
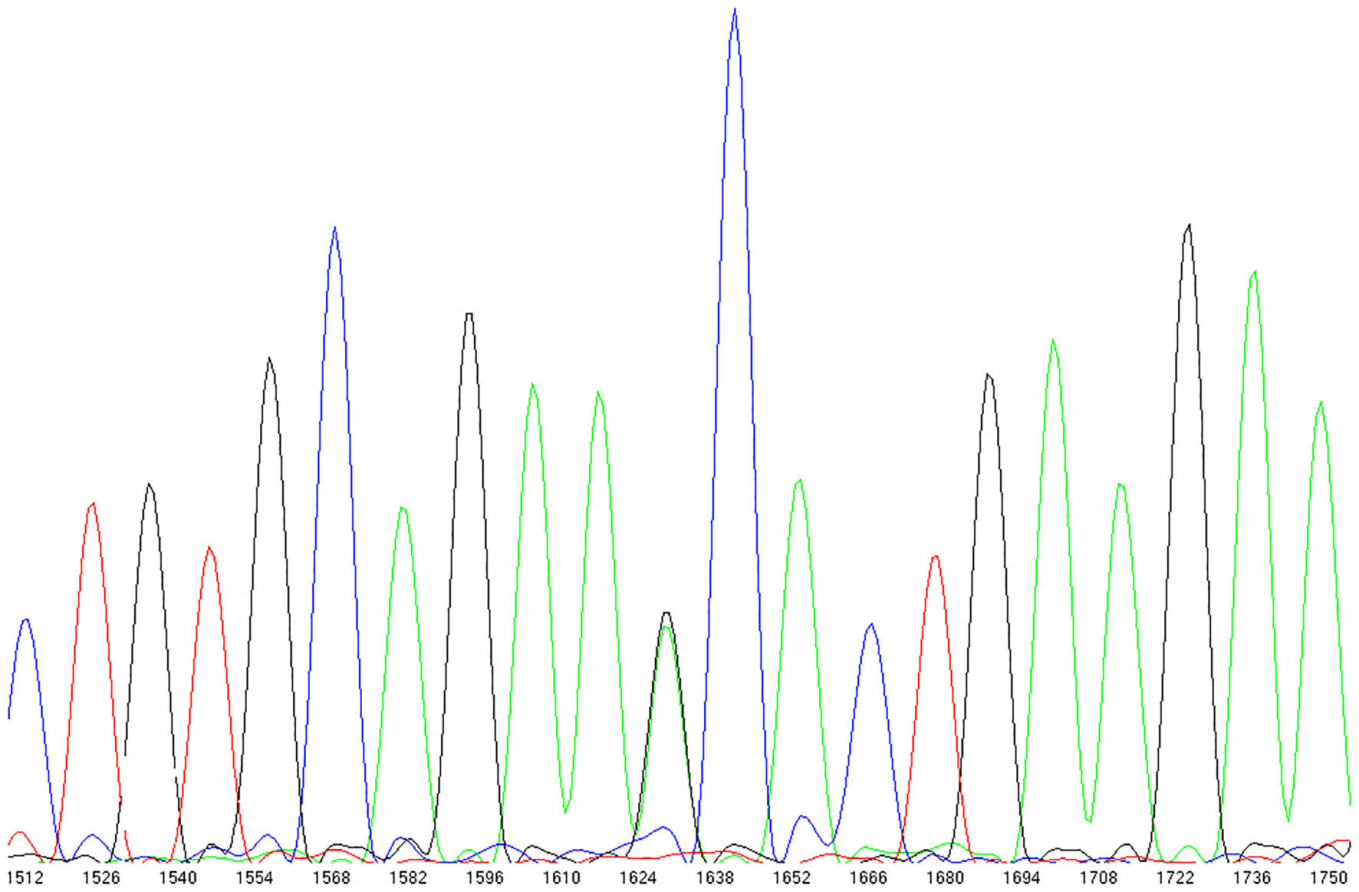
TRPC6 exon sequencing identified a rare missense variant in a breast cancer patient with chemotherapy-related heart failure. rs767086724 Asn338Ser (heterozygote) identified in 32 year old female patient (N9831 clinical trial, Arm B: sequential doxorubicin + trastuzumab) with chemotherapy-induced HF.

We observed N338S in a breast cancer patient from the N9831 clinical trial with CRHF (Figure 3). In the GnomAD browser, the 338S variant is extremely rare, observed only seven times in 141,283 individual genomes (allele frequency = 0.000025). The patient with the 338S variant was Black/African American, and in the GnomAD database the 338S allele was observed six times in 12,460 African genomes (allele frequency 0.00024), one time in 17,707 Latino genomes (allele frequency 0.000028) but not observed in other populations. In our screening sample, there were four patients with CRHF who were Black/African American (allele frequency 0.125). The N338S variant was confirmed by Sequenom genotyping and was not observed by genotyping in either control group.

### *TRPC6* Common Variants rs36111323 A404V and rs4509717 (eQTL) Are Associated With Heart Failure in Patients Who Did Not Receive Prior Anthracycline or Trastuzumab Therapy

To determine whether any of the *TRPC6* variants increased the risk of heart failure in general, we tested for association in an independent sample of 984 patients (males *N* = 433 and females *N* = 551) from the Mayo Clinic Biobank consisting of 690 cases with HF from any cause (excluding CRHF) and 282 controls who had previously received anthracycline or trastuzumab without HF. The 404V variant was significantly more frequent in patients with HF compared to controls, using an additive model (*p* = 0.002, OR 2.01) with sex and hypertension as covariates (Table 5), which remained significant at *p* < 0.05 after stringent Bonferroni correction for 16 independent tests.

**Table 5:** Association analysis of heart failure of all causes (except anthracycline or trastuzumab).

**SNP**	**Locus**	**Minor allele**	**Chemo + No HF (*N* = 282)**	**No Chemo + HF (*N* = 690)**	**Odds ratio**	***p*-value**
rs36111323	*TRPC6*	A (404V)	0.082	0.144	2.01	**0.002**
rs767086724	*TRPC6*	C (338S)	0.000	0.000	NA	NA
rs11224819	*TRPC6*	C	0.121	0.082	0.69	0.093
rs4509717	*TRPC6*	G	0.478	0.415	0.75	**0.034**
rs12280648	*TRPC6*	G	0.247	0.259	1.11	0.516
rs77679196	*TRPC6*	A	0.007	0.004	0.90	0.915
rs57242572	*TRPC6*	T	0.013	0.015	2.48	0.173
rs2513192	*TRPC6*	A	0.259	0.283	1.21	0.216
rs1938858	*TRPC6*	T	0.188	0.192	1.01	0.977
rs61918162	*TRPC6*	C	0.394	0.354	0.80	0.120
rs11224953	*TRPC6*	G	0.051	0.037	0.80	0.486
rs11224983	*TRPC6*	A	0.047	0.038	0.85	0.583
rs55756123	*LDB2*	T	0.007	0.006	1.37	0.718
rs4305714	intergenic 6p22.3	T	0.216	0.222	1.14	0.417
rs62568637	*BRINP1*	A	0.013	0.009	0.71	0.552
rs707557	*RABB22A*	T	0.399	0.431	1.17	0.251

*Association analysis was performed in 690 cases with heart failure from any cause (excluding cases ever treated with anthracycline or trastuzumab) and 282 controls who had previously received anthracycline or trastuzumab without heart failure. Minor allele frequencies are shown in each of two groups: Chemo + No HF = treated with anthracycline and/or trastuzumab and no diagnosis of heart failure; No Chemo + HF = never received anthracycline or trastuzumab and diagnosis of heart failure. SNP, single nucleotide polymorphism; NA, not applicable*.

We also observed association with a common variant, *TRPC6* rs4509717, *p* = 0.034 (Table 5). rs4509717 is an eQTL and rs4509717-A is associated with significantly higher expression of TRPC6 in cardiac tissue from both the left ventricle (*p* = 1 × 10^−5^) and tibial artery (*p* = 8.8 × 10^−8^) in the GTEX database. In our data, the A-allele is more frequent in patients with HF from all causes than in patients who received anthracycline or trastuzumab without HF.

### Inhibition of *TRPC6* Reduces Doxorubicin-Induced Apoptosis in Human iPSC-Derived Cardiomyocytes

**Figure 4:**
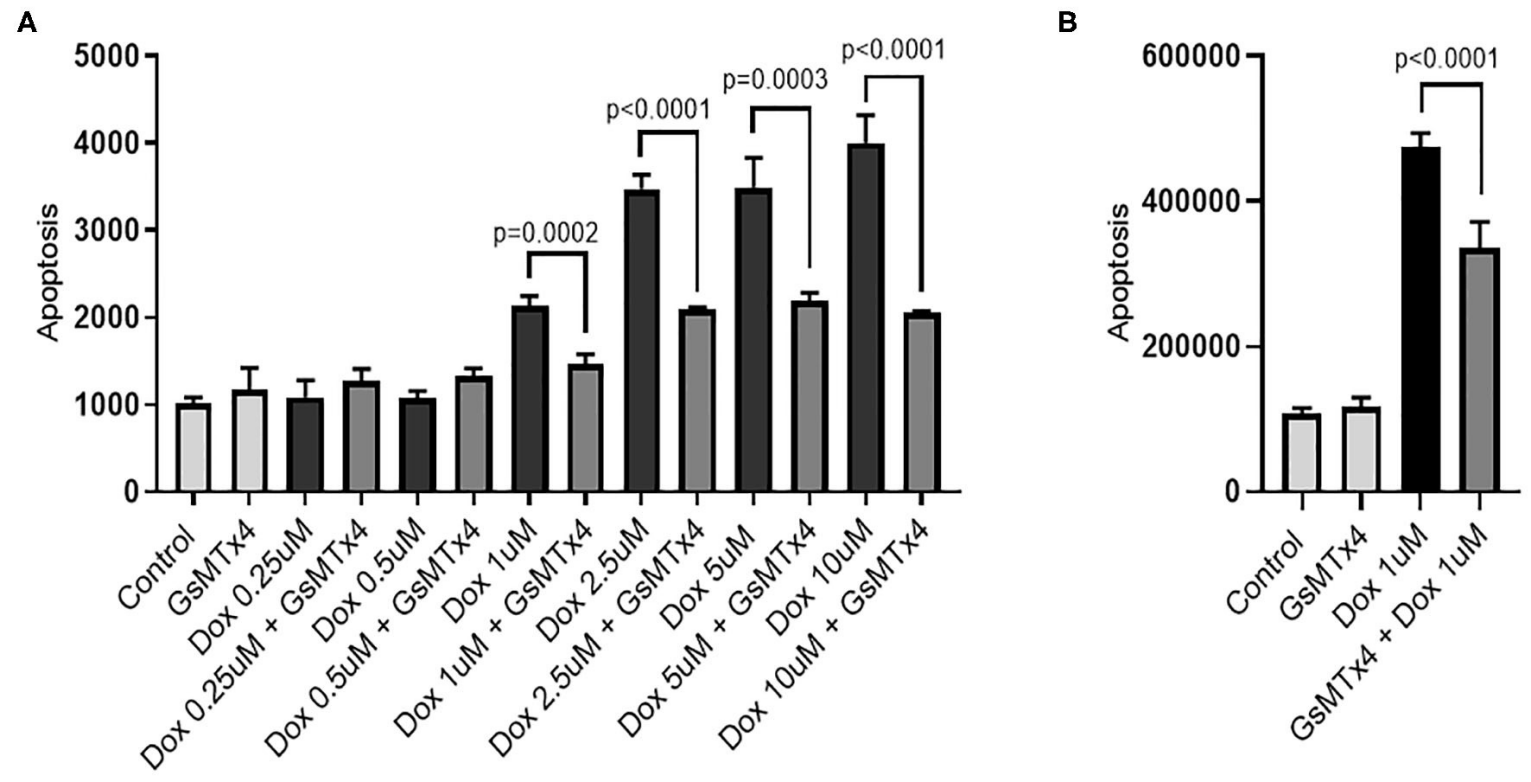
GsMTx-4 reduces doxorubicin-induced apoptosis. Apoptosis (luminescence units) in doxorubicin (Dox)-treated cells +/– GsMTx4 (5 μM) were compared by unpaired *T*-test in iPSC-derived cardiomyocytes **(A)** and iPSC-derived endothelial cells **(B)**. Error bars are standard deviation of the mean.

We used human iPSC-derived cardiomyocytes as a model to measure doxorubicin-induced apoptosis. Apoptosis increased in a dose-dependent manner (Figure 4A) as observed in humans and other cardiomyocyte models (27). We observed significant doxorubicin toxicity at 1 μM upwards. Inhibition of TRPC6 with the peptide, GsMTx-4 (28) at a final concentration of 5 μM, prior to treatment with doxorubicin at final concentrations of 1, 2.5, 5, and 10 μM significantly reduced apoptosis, *p* = 0.0002, *p* < 0.0001, *p* = 0.0003, and *p* < 0.0001, respectively (Figure 4A). Similarly, pretreatment of human iPSC-derived endothelial cells with GsMTx-4 at a final concentration of 5 μM, prior to doxorubicin at a final concentration of 1 μM, significantly reduced apoptosis, *p* < 0.0001 (Figure 4B). GsMTx-4 (5 μM) alone had little/no effect on apoptosis in either cell type.

### Inhibition of TRPC6 Prevents Doxorubicin-Induced Cardiac Dysfunction in an Animal Model of Doxorubicin-Induced Cardiomyopathy

**Figure 5:**
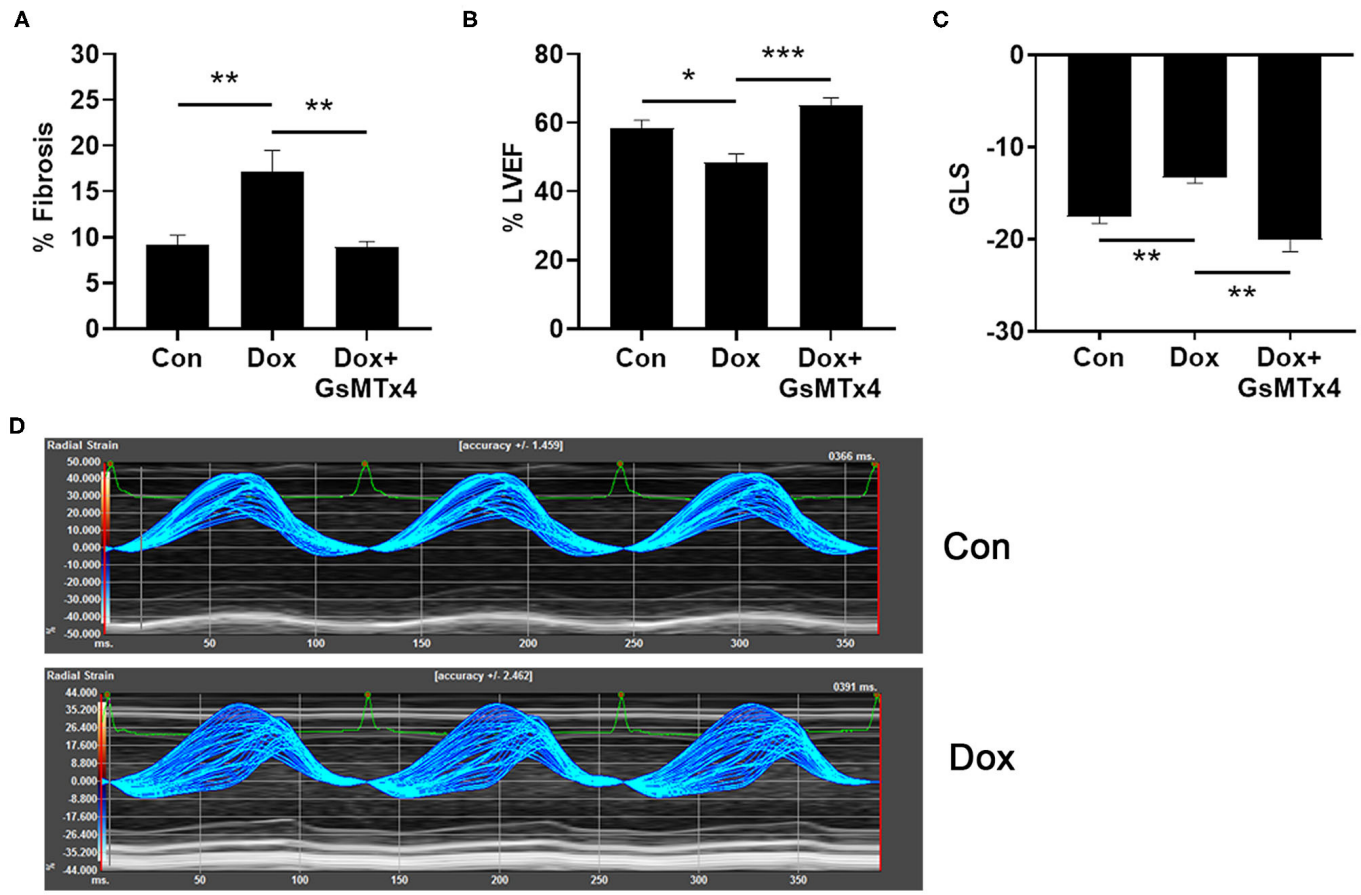
Doxorubicin causes fibrosis and cardiomyopathy that is prevented by Trpc6 inhibition. Male B6.129 WT mice were injected intraperitoneally (ip) with doxorubicin (Dox) on days 1, 3, 5, 8, 10, 12 (cumulative dose 24 mg/kg) ± the Trpc6 inhibitor GsMTx-4 (10 mg/kg) or saline/DMSO control (Con) ip during Dox therapy and echocardiography-derived LVEF and global longitudinal strain (GLS) performed at day 21. **(A)** Dox-induced fibrosis was prevented in mice that were pre-treated with GsMTx-4. 1-Way ANOVA test revealed significant differences between the 3 groups, *p* = 0.0005. **(B)** Dox-induced decrease in LVEF was prevented in mice that were pre-treated with GsMTx-4. 1-Way ANOVA test revealed significant differences between the 3 groups, *p* = 0.0004. **(C)** GLS measures of mice in Con and Dox-treated groups were comparable to normal function and impaired cardiac function in humans, respectively. Dox-induced increase in GLS was prevented in mice that were pre-treated with GsMTx4. 1-Way Kruskal-Wallace test revealed significant differences between the 3 groups, *p* = 0.0006. **(D)** Parametric maps of radial strain of the endocardium through 3 cardiac cycles. The larger spread of the lines during systole in a representative wild type Dox male mouse depicts less coordinated contraction (i.e., cardiac dysfunction) compared to a wild type Con mouse. Data are shown as mean ± SEM, ^*^p < 0.05; ^**^p < 0.01; ^***^p < 0.001 using unpaired test. Multiple-comparisons of groups was performed using Mann-Whitney or Sidak tests with a Bonferroni adjusted *p*-value for 3 independent tests of <0.017 regarded as significant.

In order to determine whether inhibition of TRPC6 with the peptide GsMTx-4 could prevent cardiomyopathy induced by doxorubicin in an animal model, we injected wild type B6.129 male mice with 6 doses of 4 mg/kg doxorubicin or saline/DMSO control intraperitoneally (ip) on days 1, 3, 5, 8, 10, 12 (cumulative dose of 24 mg/kg), and performed echocardiography at day 21 to determine cardiomyopathy and histology to measure cardiac fibrosis. As expected, fibrosis was significantly worse in mice that had received doxorubicin at day 21 compared to controls, *p* = 0.0014, but was prevented in mice that were pre-treated with GsMTx-4 (*p* = 0.0014) (Figure 5A). Cardiac function was significantly worse in mice that had received doxorubicin at day 21 assessed by echocardiography (LVEF, *p* = 0.017) (Figure 5B) and echocardiography-derived global longitudinal strain (GLS) (*p* = 0.004) (Figure 5C), which measures regional changes in cardiac muscle function (Figure 5D) (20). In contrast, LVEF and GLS were preserved in mice that received treatment with the TRPC6 inhibitor GsMTx-4 in addition to doxorubicin (*p* = 0.0003 and *p* = 0.002, respectively) (Figures 5B,C). Significant differences were observed between the 3 groups for fibrosis, LVEF and GLS with *p*-values of 0.0005, 0.0004, and 0.0006, respectively. Generally, global longitudinal strain values of < -14% (negative and thus closer to 0) are considered pathogenic and values of about −18% are considered normal (20).

## Discussion

Predictors of anthracycline and trastuzumab cardiotoxicity including genetic “risk” variants are lacking in the clinical setting. To date several GWAS studies of anthracycline cardiotoxicity have been published (29–31) including our own GWAS of cardiotoxicity in patients treated with anthracycline +/– trastuzumab (12). At this time, there is little overlap between the top associated loci in each study. Further barriers to integrate genetic markers into the clinical setting include the definition of cardiotoxicity. This is largely because the proportion of patients who experience anthracycline or trastuzumab-related CHF is relatively small, resulting in a lack of statistical power when using this most important and extreme phenotype. An alternative approach has been to use the maximum decline in LVEF as a quantitative indicator of cardiomyopathy (12, 31), but this approach can also be problematic due to the variability inherent in echocardiography, and that a significant proportion of patients with a measurable decline in LVEF do not progress to symptomatic CHF.

We previously published a GWAS of decline in LVEF in early breast cancer patients treated with doxorubicin and paclitaxel +/– trastuzumab (12). GWAS of CHF as a binary phenotype was considered not appropriate at the time, because there were only 17 patients with cardiologist confirmed CHF. Therefore, in this study we focused on replication of the loci at *p* < 1 × 10^−5^ using two different replication strategies and functional validation in human iPSC-derived cardiomyocytes and endothelial cells. These studies identified the cation channel, *TRPC6* as a risk locus for chemotherapy-related cardiotoxicity using both decline in LVEF and CHF as phenotypes, and preliminary evidence for *TRPC6* as a therapeutic target for protection against doxorubicin-induced cardiotoxicity. Suggestive, but lesser evidence was also observed for the adapter molecule LIM-domain binding 2, *LDB2*.

In the first replication strategy, we used an independent dataset of genotype and gene expression levels from LV cardiac tissue from the GTEX database to impute gene expression levels in our primary GWAS dataset and tested for association of decline in LVEF with imputed gene expression levels. Of the top six GWAS loci, only four mapped within coding genes (*TRPC6, RAB22A, LDB2, BRINP1*). Of the four coding loci, only *TRPC6* gene expression was imputed in cardiac tissue, and associated with a decline in LVEF (*p* = 0.005). These data indirectly suggest that non-coding genetic variants influence *TRPC6* expression in the heart increasing the risk of CRHF and are supported by two independent lines of evidence. First, there are previous studies showing drug susceptibility SNPs for six different chemotherapies including the anthracycline, daunorubicin (32–38), and these SNPs have been shown to be significantly more likely to be located in eQTL loci (39). Second, animal studies have demonstrated that overexpression of *Trpc6* leads to hypertrophic cardiomyopathy and heart failure in mice (40) and genetic deletion or inhibition of *Trpc6* reduces pressure overload in models of HF (41). We note here, in the study of Seo et al. (41), deletion of Trpc6 was only protective against cardiac remodeling in combination with deletion of Trpc3 and two other studies using pressure overload to model HF in mice have identified Trpc3 as a driver of maladaptive cardiac remodeling (42) and fibrosis (43). It is known that both TRPC3 and TRPC6 can assemble into either homotetramers or heterotetramers with each other (44), but it is not known how changes in TRPC6 expression relate to changes in the ratio of homo/heterotetramerisation in the heart (or vascular tissues) and what the functional consequences could be. A study of the pathophysiological roles of Trpc3 and Trpc6 in vascular smooth muscle cells demonstrated that unlike Trpc6 channels, Trpc3 channels are constitutively active as determined by TRPC-carried cation currents, and that Trpc3 channels are upregulated in Trpc6 deficient cells, but are not able to functionally replace Trpc6 channels (45). Therefore, while our data show association of genetic variants that correlate with TRPC6 expression, their pathophysiological effects on TRPC6 channel function and interaction with other TRPC channels in humans are likely complex.

Our second replication approach of the GWAS loci assessed association with CHF as a binary phenotype in the context of control groups of patients treated with chemotherapy and trastuzumab with no HF and patients with HF who were never treated with any type of anthracycline or trastuzumab. Due to this selection of controls, the characteristics of each group were different from each other in terms of demographics and cancer characteristics. We made the assumption that risk variants for CRHF would be more frequent in cases with chemotherapy-related CHF than in both of the two control groups. These analyses further suggested that variants associated with *TRPC6* expression levels in the heart are potential risk variants for CRHF. The variant rs57242572 in the *TRPC6* 5′ flanking region, significant in the discovery GWAS and a common eQTL variant for *TRPC6* in the left ventricle (rs61918162, not genotyped in our initial GWAS), were associated with CRHF in the Mayo Clinic Biobank analysis. The rs61918162-T allele was strongly associated with higher *TRPC6* expression in heart tissue in the GTEX database and this same allele was more frequent in patients with CRHF. Taken together, these associations further suggest that variants that increase *TRPC6* expression in the heart are risk factors for CRHF.

We also replicated the association of the sentinel GWAS SNP in *LDB2*. This variant rs55756123, which is located in the 3′ flanking region, was not listed as an eQTL in the GTEx database although this may be an issue of statistical power due to the relatively low MAF (~0.02 in European ancestry). We believe this locus warrants additional investigation.

To further explore *TRPC6*, and because our initial GWAS was not powered to interrogate rare missense mutations, we also screened the exons of *TRPC6* for rare missense mutations in 38 patients with chemotherapy-related CHF. We observed one very rare missense variant (N338S) in an African American patient from the N9831 trial who received doxorubicin, paclitaxel, and trastuzumab and was deemed to be at low risk for cardiotoxicity based on published risk factors of sex, older age, and low LVEF prior to chemotherapy (1, 5). The 338S variant was observed in 1/4 African American patients in our screening set, ~400-fold enrichment compared to the African population in GnomAD. Twenty one missense variants in *TRPC6* have been identified in patients with the genetic form of chronic kidney disease FSGS, (25) most of which are deemed gain-of-function by measurement of calcium influx. The N338S variant in our study is not reported in FSGS patients, hence there are no published functional data relative to increased calcium influx for this variant. N338S is determined as probably damaging by Polyphen, and maps between the linker helices 5 and 6 of *TRPC6*, a region for which little is known, and no known gain-of-function mutations to date map to this region. Therefore, it is difficult to determine if this is a chance finding, or that perhaps very rare missense variants could increase patient risk of CRHF.

Finally, our data suggest that patients with the *TRPC6* 404V allele and the rs4509717-A allele (associated with higher *TRPC6* expression) are at greater risk of HF in general. Under our experimental design and assumptions, the common *TRPC6* missense variant, A404V, was not associated specifically with CRHF, although rs4509717 did show a trend (*p* = 0.09). 404V has previously been identified in patients with FSGS, but was always discounted as disease causing because the incidence rates of FSGS range from 0.2 to 1.8 per 100,000 individuals (46) and the 404V allele frequency in Europeans (as per gnomAD) is 0.12. However, the 404V allele is predicted to be “probably damaging” by Polyphen (47) and results in increased calcium influx relative to 404A, consistent with a gain-of-function phenotype (25). In our studies 404V was associated with both maximum decline in LVEF in our initial GWAS and with HF of all causes in the Mayo Clinic Biobank patients, with the largest effect size under a recessive model. To our knowledge *TRPC6* has not previously been identified as a risk locus in published GWAS studies of HF, although there is a single report of *TRPC6* associated with diastolic blood pressure (48), in which rs61892344-T is associated with both diastolic blood pressure and higher *TRPC6* expression in the tibial artery (*p* = 2.6 × 10^−17^) and artery from aorta (*p* = 2.9 × 10^−6^).

We next sought to test TRPC6 as a potential therapeutic target for cardio-protection in patients treated with doxorubicin by inhibition with GsMTx-4. GsMTx-4 is a peptide found in the venom of the Chilean rose tarantula (*Grammostola spatulata*) (49) and was shown by Spassova et al. to block the activation of TRPC6 channels by either receptor-induced PLC activation or by direct activation of diacylglycerol (28). Our TWAS experiment showed association of TRPC6 imputed gene expression in the left ventricle with a decline in LVEF, hence we began this experiment in human iPSC-derived cardiomyocytes. However, given that TRPC6 is more highly expressed in vascular tissue than in the heart (50), (an important consideration for functional validation) and doxorubicin cardiotoxicity may also be mediated through endothelial dysfunction (51), we also performed the experiment in human endothelial cells. Pre-treatment of both cell types with GsMTx-4 significantly reduced doxorubicin-induced apoptosis, suggesting that inhibition of TRPC6 may reduce cardiotoxicity in patients receiving doxorubicin. Next, we sought to prevent cardiomyopathy caused by doxorubicin using GsMTx-4 in an animal model and found blocking TRPC6 *in vivo* significantly improved cardiac remodeling/fibrosis and dysfunction caused by doxorubicin. These data suggest that inhibition of TRPC6 may be a therapeutic option for patients with genetic susceptibility that undergo anthracyclines.

In summary, our data suggest that *TRPC6* is a risk locus for both CRHF and HF in general. In line with animal models where higher *TRPC6* expression led to pathologic remodeling, hypertrophic cardiomyopathy, and heart failure (40) our study identified genetic variants that were associated with higher *TRPC6* cardiac expression that occurred more frequently in patients with CRHF and in patients with HF of all causes. Importantly, blocking *TRPC6 in vitro* and *in vivo* reduced doxorubicin-mediated toxicity and cardiomyopathy further suggesting that inhibition of *TRPC6* may have potential as a cardioprotective therapy. Finally, we identified a very rare missense variant in *TRPC6* that could increase risk for CRHF in patients of African ancestry and a common missense variant with prior functional evidence for increased calcium influx that may also be useful to build into a risk model for cardio-oncology practice.

## Limitations

We used a transcriptome-wide association study (TWAS) to extract further information from our initial GWAS, which in itself has some caveats. First and most obviously, gene expression levels of patients in N9831 were imputed and not obtained from actual patient heart tissue. Validation of actual gene and/or protein expression is required. Furthermore, the imputed gene expression levels are the estimated “heritable component” of gene expression, not total expression. Secondly, our TWAS analysis identified 22 other genes that were more significantly associated with decline in LVEF than *TRPC6*, but follow-up of those genes was beyond the scope of this study.

Our replication studies of the top GWAS loci using a binary phenotype of CHRF were limited by the small sample size of 38 cases, which did not allow for analysis by trastuzumab status and included 17 cases from the primary GWAS study of decline in LVEF, and are therefore, not truly independent. Other limitations are the definitions of HF based on the eMERGE algorithm for the Biobank samples, because is it possible that control patients with chemotherapy and no HF did have an asymptomatic decline in LVEF at some point. In the second control group, 57% of patients had a cancer diagnosis prior to HF. Therefore, although they had never received anthracycline or trastuzumab, some of the patients in this group could have HF resulting from other cancer treatments. What we can say from this study design is that comparison of CRHF cases to a control group that included patients with HF from other cancer therapies would weaken the reported association rather than cause a false positive association, and that the observed genetic associations relate specifically to anthracycline and/or trastuzumab related HF. Further work is needed to assess the functional consequences of the associated genetic variants and to elucidate the molecular mechanism of *TRPC6* relative to doxorubicin-induced cardiomyopathy and HF in general.

Finally, there are several known inhibitors of *TRPC6*, all with different specificities, some of which also inhibit other members of the TRPC family. *TRPC6* knock-down or knock-out animal models will be required to demonstrate that inhibition of *TRPC6* alone prevents or reduces doxorubicin-induced cardiotoxicity and cardiomyopathy. We were also unable to test whether inhibition of *TRPC6* prevented trastuzumab-related cardiotoxicity using an iPSC-derived cardiomyocyte cell culture model because in our hands, no clinically relevant amount of trastuzumab or length of time induced cardiomyocyte apoptosis (data not shown). We were also unable to test LDB2 as a potential therapeutic target due to lack of available inhibitors.

## Conclusions

Genetic variants that are associated with increased *TRPC6* expression in the heart and *TRPC6* missense variants may be clinically useful as biomarkers for CRHF, but further replication in larger samples is required. GsMTx-4 may be able to prevent or reduce doxorubicin-induced cardiotoxicity and cardiomyopathy in patients with increased susceptibility. Further functional validation is required to determine if TRPC6 inhibition might be more relevant as a therapy for patients with specific *TRPC6* risk variants. As a future direction, several known inhibitors of *TRPC6* and other TRPC family members may be harnessed to determine their therapeutic relevance for doxorubicin-induced CHF.

## Data Availability Statement

The original contributions presented in the study are included in the article/Supplementary Material, further inquiries can be directed to the corresponding author/s. The names of the repository/repositories and accession number(s) from which publicly accessible data was harvested for the purposes of the study can be found in the article/Supplementary Material.

## Ethics Statement

The studies involving human participants were reviewed and approved by Mayo Clinic Institutional Review Board. The patients/participants provided their written informed consent to participate in this study. The animal study was reviewed and approved by Mayo Clinic IACUC.

## Author Contributions

NN designed the genetic and cell culture experiments, analyzed, and interpreted the data and wrote the manuscript. JC performed statistical analyses. DS performed the TWAS analysis. BN and PB performed cell-based assays. LW and JO extracted and annotated data from the Mayo Clinic Biobank. JK performed DNA sequencing. PA, JR, and CL reviewed clinical data. DD, AH, KB, and DF designed, performed, analyzed, and interpreted animal experiments. DF reviewed and interpreted the data and assisted with writing the manuscript. All authors critically revised the manuscript.

## Conflict of Interest

The authors declare that the research was conducted in the absence of any commercial or financial relationships that could be construed as a potential conflict of interest.

## Supplementary Material

Supplementary Table 1 Gene-based association analysis of imputed gene expression in heart left ventricle and maximum decline in LVEF in 800 breast cancer patients treated with doxorubicin, paclitaxel and trastuzumab identified from the N9831 study. PrediXcan analysis using adjusted linear models on imputed expression in heart left ventricle, beta values were scaled based on the range of output expression values for each gene to represent the average change in LVEF per each standard deviation in expression. For example, *TRPC6* has an adjusted beta of −0.65, such that an increase of one standard deviation in *TRPC6* expression is associated with a decrease of 0.65 in the maximum drop of LVEF. L95, 95th% lower confidence limit; U95, 95th% upper confidence limit.

Supplementary Table 2 Genotype counts for each SNP in each of group of controls and cases with chemotherapy-related congestive heart failure.
